# Cardiovascular Mechanisms of Exercise Intolerance in Older Patients with Heart Failure

**DOI:** 10.31083/j.rcm2309313

**Published:** 2022-09-13

**Authors:** Narayana Sarma V. Singam, Meir Tabi, Jerome L. Fleg

**Affiliations:** ^1^Division of Critical Care Medicine, Mayo Clinic, Rochester, MN 55903, USA; ^2^Department of Critical Care, Washington Hospital Center Washington DC, 20010, USA; ^3^Division of Cardiology, Washington Hospital Center, Washington DC, 20010, USA; ^4^Department of Cardiology, Mayo Clinic Rochester, MN 55903, USA; ^5^Division of Cardiovascular Sciences, National Heart, Lung, and Blood Institute, Bethesda, MD 20892, USA

**Keywords:** elderly patient, heart failure, exercise intolerance, peak oxygen consumption

## Abstract

Exercise intolerance, measured by peak oxygen consumption (V̇O2), is a hallmark feature of heart failure (HF). The effect is compounded in the elderly HF patient by aging-associated changes such as a reduction in lean muscle mass, an increase in adiposity, and a reduction in maximal heart rate and peripheral blood flow with exercise. There is a non-linear reduction in peak V̇O2 with age that accelerates in the later decades of life. Peak V̇O2 is further reduced due to central and peripheral maladaptation from HF. Central mechanisms include impaired peak heart rate, stroke volume, contractility, increased filling pressures, and a blunted vasodilatory response. Peripheral mechanisms include endothelial dysfunction, reduced blood flow to muscles, and impaired skeletal muscle oxidative capacity. This review presents a focused update on mechanisms leading to impaired aerobic capacity in older HF patients.

## 1. Background

Heart failure (HF) is prevalent in the elderly and exceeds 10% in those older than 85 years [1]. Over 75% of HF cases involve older adults [2]. At least half of these cases involve HF with preserved ejection fraction (HFpEF) [3]. Regardless of left ventricular ejection fraction (LVEF), a hallmark feature of HF is exercise intolerance, as demonstrated by a reduction in peak oxygen consumption (V̇O2) with exercise [4]. Elderly patients are also most vulnerable to complications associated with HF. Patients with a low peak V̇O2 are at an increased risk for mortality.

This review presents a focused update of the cardiovascular and peripheral processes leading to exercise intolerance in older HF patients.

## 2. Peak V̇O2 as a Measure of Aerobic Exercise Capacity

Peak oxygen consumption (V̇O2) is considered the “gold standard” for measuring aerobic performance. It is a product of cardiac output (CO), the central component, and the arterio-venous oxygenation difference (A-V̇O2 diff), the peripheral component, as described by the Fick equations below,

Equations:

(1) $$\begin{array}{c} V\,O\,2=C\,O\times A\,V\,O_{2}\,\text{ diff, }\\ V\,O\,2=S\,V\times H\,R\times A\,V\,O_{2}\,\text{ diff, }\\ V\,O\,2=E\,D\,V\times L\,V\,E\,F\times H\,R\times {\mathrm{AVO}}_{2}\,\text{ diff, } \end{array}$$

V̇O2, peak oxygen consumption; CO, cardiac output; EDV, end-diastolic volume; LVEF, left ventricular ejection fraction; AV$O_{2}$, arteriovenous oxygen difference; SV, stroke volume; HR, heart rate.

**Fig. 1.**
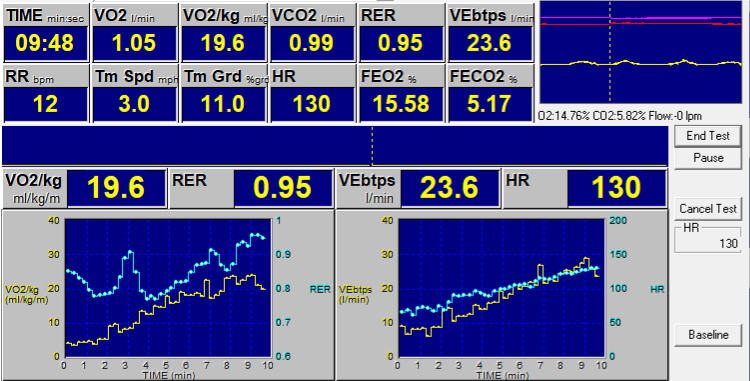
**Example of a cardiopulmonary exercise test (CPET) report**. Oxygen consumption (VO2) per kilogram of body weight (left) and volume of expired air (VE) (right) are plotted over time in minutes. BTPS, body temperature; pressure; saturated water vapor; FEO2%, Concentration of oxygen in exhaled gasses; FECO2%, Concentration of carbon dioxide in exhaled gasses; HR, heart rate; RER, Respiratory exchange ratio; Tm Grd, treadmill grade; Tm Spd, treadmill speed.

Mechanisms that alter any of the variables of the equations may affect aerobic performance. Peak V̇O2 is measured by cardiopulmonary exercise testing (CPET) to evaluate functional capacity. The examination is usually performed with a cycle ergometer or a treadmill. The patient’s heart rate and blood pressure and electrocardiogram (ECG) are continuously recorded while expired gasses (i.e., oxygen and carbon dioxide) are analyzed. Measurements are obtained at rest, throughout exercise, and during recovery. The V̇O2 is plotted as a function of time and correlates with the patient’s work [5]. Fig. 1 demonstrates a sample plot of V̇O2 and other exercise variables versus time. Both central and peripheral determinants are responsible for a blunted peak V̇O2 with exercise in the elderly HF population by affecting one or more of the parameters in the equations. The processes involving each variable are described below.

### 2.1 Effects of Age and Gender on Peak V̇O2

Peak V̇O2 (i.e., exercise capacity) is inversely correlated with age in both cross-sectional and longitudinal studies [6, 7]. Fleg and colleagues [7] evaluated the longitudinal change in peak V̇O2 of healthy volunteers from the Baltimore Longitudinal Study of Aging (BLSA) cohort over eight years. This study demonstrated a steep reduction in peak V̇O2 as a function of increased age regardless of gender. The rate of peak V̇O2 reduction was higher in later decades (>70-years-old) and exceeded 20% per decade in such individuals. Two additional studies have since confirmed these findings [8, 9]. Although aerobically active individuals maintain a higher peak exercise capacity than their sedentary counterparts, they experience similar relative reductions in peak V̇O2 with increased age [7, 8].

The age-related decline in peak V̇O2 is a result of several factors. The decrease in maximal heart rate of approximately one beat per minute per year is a major contributor to this reduction in peak V̇O2 by its effect on exercise CO. This decline in maximal heart rate with age is likely mediated by a reduction in beta-adrenergic responsiveness, which has been demonstrated by blunted heart rate increase from infused catecholamines [10]. Age-related decreases in O2 pulse (i.e., the product of stroke volume and A-V̇O2 difference) also correlate well with peak V̇O2 changes and suggest underlying peripheral factors also influence exercise capacity [7]. These factors include loss of lean body mass [7], reductions in blood flow to muscles [11], impaired muscular oxidative metabolism [12], increased arterial wall stiffness [13], and reduced peripheral oxygen extraction [14]. Table 1 (Ref. [6, 15, 16, 17, 18, 19, 20, 21, 22, 23, 24, 25, 26, 27, 28, 29]) summarizes the mechanisms responsible for reducing aerobic capacity with aging.

**Table 1.** **Aging and heart-failure related mechanisms for changes in aerobic capacity**.

		Aging	Heart failure
Peak V̇O2		↓	↓
Central Mechanisms			
	Maximal SV	↓/=	↓
	Peak HR	↓	↓
	Peak CO/CI	↓	↓
	Maximal LV EDV	↑	↑
	Maximal EF	↓	↓
	Diastolic function	↓	↓
Peripheral Mechanisms			
	Maximal A- V̇O2 diff	↓	↓
	Peak SVR/SVRI	↑	↑
	Lean muscle mass	↓	↓
	Mitochondrial volume/function	↓	↓
	Peripheral blood flow	↓	=/↓

A summary of age and heart failure related mechanisms for changes in aerobic capacity. Summarized from citations [6, 15, 16, 17, 18, 19, 20, 21, 22, 23, 24, 25, 26, 27, 28, 29]. A-V̇O2 diff, Arterio-venous oxygen concentration difference; CI, cardiac index; CO, cardiac output; EF, ejection fraction; EDV, end diastolic volume; HR, heart rate; LV, left ventricle; SV, stroke volume; SVR, systemic vascular resistance, SVRI, systemic vascular resistance index; SVR, systemic vascular index; V̇O2, oxygen consumption.

There are also sex differences in peak V̇O2 across the age span, with women demonstrating values approximately 20% lower than men. The sex difference is primarily related to the smaller muscle mass in women [30]. In healthy BLSA volunteers, there was a mean 44% reduction in peak V̇O2 in men and a 36% decline in women between ages 25 and 75 years [7].

## 3. Heart Failure and Its Relation to Peak V̇O2 in the Elderly

Heart failure with reduced ejection fraction (HFrEF) is defined by a reduction in the LVEF below 40%, and HFpEF by an LVEF ≥45% or 50% in association with the classic HF symptoms of dyspnea, fatigue, and exercise intolerance. The elderly are already at risk for an age-related reduction in maximal exercise capacity due to the processes mentioned in the previous section. Heart failure (regardless of LVEF) is an independent risk factor for further exercise intolerance as measured by a reduction in peak V̇O2 compared to healthy age peers [30].

In one study, older patients with HF (mean age: 70 years) demonstrated a blunted peak V̇O2 during upright cycle ergometry: (HFrEF: 13.1 mL/kg/min, HFpEF: 14.2 mL/kg/min) compared to similarly aged healthy controls: 19.9 mL/kg/min [31]. A subgroup analysis of the participants in H*eart Failure: A Controlled Trial Investigating Outcomes of Exercise Training (*HF-ACTION) study demonstrated that age is the strongest predictor of peak V̇O2 in HFrEF patients [32]. This study showed a reduction of peak V̇O2 by approximately 1 mL/kg/min for every 7-year increase in the age above 40 years.

Lower peak V̇O2 is a potent risk factor for adverse outcomes in older HF patients similar to younger cohorts. A 2015 large multi-center prospective study evaluating 990 elderly (≥70 years old) patients with HFrEF determined that higher peak V̇O2 was predictive of reduced risk of cardiovascular death or urgent heart transplant (hazard ratio: 0.97, *p* = 0.0016) [33]. This study demonstrated a graded reduction in median peak V̇O2 in mL/kg/min with each decade of life: <50 years: 17.1 mL/kg/min, 50 to <60 years: 14.8 mL/kg/m, 60 to <70 years: 13.9 mL/kg/min, and ≥70: 12.5 mL/kg/min. A peak V̇O2 less than 14 mL/kg/min in HFrEF patients is commonly used as a major criterion for cardiac transplantation referral [34].

Compared to patients with HFrEF, patients with HFpEF tend to be older, more often female, and have more comorbidities, such as obesity, diabetes mellitus, and hypertension [35]. Blunted exercise tolerance and impaired peak V̇O2 are also characteristic features of HFpEF patients. Haykowsky and colleagues [36] compared the peak V̇O2 of 60 older HFpEF patients (mean age: 70 years) undergoing CPET to age-matched healthy control subjects (N: 40, mean age: 69 years). They demonstrated a significantly reduced peak V̇O2 in the HFpEF patients compared to control subjects (cycle ergometer peak V̇O2: 14.6 vs. 22.9 mL/kg/min, respectively) [36]. Multiple studies have corroborated these results [15, 16, 37, 17]. Although studies relating mortality to exercise intolerance in elderly HFpEF patients are less common than in HFrEF, data in younger patients with HFpEF indicate that peak V̇O2 is similarly predictive of mortality [38]. A study by Yan and colleagues demonstrated that increased minute ventilation to carbon dioxide production (VE/VCO2) slope, a marker of excessive ventilation for work performed, was more predictive of mortality compared to peak V̇O2 alone in 224 older patients (mean age 69 years) with HFpEF [39]; however, more studies are needed to validate these results.

Peak V̇O2 is reduced with exercise in HF patients secondary to derangements in multiple central (i.e., heart rate, contractility, ventricular relaxation) and peripheral (i.e., skeletal muscle volume/function, metabolism, vasodilator reserve) parameters. The following sections will review these mechanisms in detail. Table 1 delineates the mechanisms for changes in aerobic capacity in older HF patients. The similarity of these changes to those related to aging per se is striking, providing a “double dose” via their superimposition on the aging changes.

## 4. Central Mechanisms of Exercise Intolerance in Elderly HF Patients

A reduction in peak CO between 27% and 58% is notable in patients with HF compared to healthy individuals of similar age [40]. Understanding the mechanisms leading to impaired CO, the product of heart rate and stroke volume, is key in recognizing the central processes leading to blunted peak V̇O2 response with exercise.

### 4.1 Heart Rate and Stroke Volume 

Impaired peak CO in patients with HFrEF is commonly due to both heart rate and stroke volume reductions. Chronotropic incompetence (CI) with exercise, as defined by a reduced ability to augment heart rate response to exercise, is common in HF [41]. A 2006 study by Brubaker and colleagues [42] compared the heart rate response to upright cycle ergometry in 102 older patients (>65 years) with HFrEF and age-matched control subjects. Approximately 22% of the HF group demonstrated CI, as defined by failure to achieve at least 80% of age-predicted maximal heart rate on the exercise test despite maximal effort. This parameter correlated with lower peak V̇O2 (12.4 mL/kg/min) as compared to HFrEF patients without CI (peak V̇O2: 14.6 mL/kg/min) and healthy controls (peak V̇O2: 19.1 mL/kg/min). These data suggest that a blunted heart rate response plays a major role in the impaired CO response to exercise and is a key mechanism for exercise intolerance in HF. It is well recognized that stroke volume augmentation is also blunted with exercise by approximately 50% in patients with HFrEF [43, 44, 45].

Chronotropic incompetence and impaired stroke volume responses are also evident in elderly HFpEF patients. A clinical trial by Borlaug and colleagues demonstrated a significantly blunted heart rate response to upright cycle ergometry in elderly HFpEF patients (mean age: 65 years; mean baseline heart rate: 70 bpm, mean peak heart rate: 87) as compared to control subjects (mean age: 65 years, mean baseline heart rate: 68 bpm, mean peak heart rate: 115 bpm) [17]. The HFpEF patients also demonstrated a slower heart rate recovery, although there was no difference in atrioventricular nodal blockade agent use between groups. A 2011 study by Haykowsky and colleagues [18] evaluated 48 elderly HFpEF patients (mean age: 69 years) and also demonstrated that a reduced peak V̇O2 resulted from a lower CO, primarily due to a blunted response in peak heart rate. In this study, stroke volume augmentation was preserved, contrary to their previous study [46]. However, a recent study demonstrated an impaired stroke volume response to exercise in older HFpEF patients [47].

### 4.2 Impaired Cardiac Contractility 

Impaired cardiac contractility is a hallmark feature of HFrEF and is characterized by a reduction in LVEF. LVEF may be further compromised due to excessive vasoconstriction in an elevated afterload state. An impaired contractile reserve is also noted in elderly HFpEF patients. Borlaug and colleagues demonstrated a reduction in peak power index, defined by the product of peak LVEF and systolic blood pressure divided by end-diastolic LV volume, and end-systolic elastance, defined by a ratio of end-systolic LV pressure to end-systolic LV volume, in 17 HFpEF patients (mean age: 65 years) compared to healthy controls [17].

### 4.3 Impaired Left Ventricular Relaxation and Increased Filling Pressures

Regardless of LVEF, patients with HF have impaired left ventricular relaxation and increased filling pressures at a reduced workload compared to their healthy counterparts [19, 48, 49, 50]. Maeder and colleagues [19] demonstrated increased pulmonary capillary wedge (PCWP) pressures in elderly HFpEF patients at a lower workload when compared to healthy controls. The rapid rise of PCWP with exercise suggests decreased left atrial and ventricular compliance from impaired lusitropy, contributing to poor aerobic performance in these patients [19, 49]. This finding is also notable in patients with HFrEF. In 2012, Sandri and colleagues demonstrated that patients with HFrEF, including those ≥65 years old, also show significant diastolic dysfunction [48]. The resultant effect is elevated left ventricular and atrial filling pressures, and increased mitral regurgitation, contributing to exercise intolerance [50].

## 5. Peripheral Mechanisms of Exercise Intolerance in Elderly Patients

Several significant peripheral structural, functional, and metabolic abnormalities lead to exercise intolerance in patients with HF [43, 51]. The elderly are more susceptible to these effects for the following reasons: reduced skeletal muscle mass, alterations in peripheral muscle composition and function, increased sedentary lifestyle, more comorbidities (i.e., arthritis, diabetes, hypertension), and impairments to metabolism [30]. Several recent trials in the elderly demonstrate significant peripheral alterations in patients with both HFrEF and HFpEF and are described below.

### 5.1 Skeletal Architecture Variations 

Exercise training improves exercise capacity despite limited effects on CO, stroke volume, and left ventricular stiffness in older patients with HF [52, 53]. The likely explanation is that peripheral maladaptations are crucial contributors to exercise intolerance in HF via reduction in arteriovenous (A-V̇O2) oxygen difference, an essential reflection of skeletal muscle architecture and function [54]. An increase in fat mass, seen both with aging and obesity, is also a contributor to reduced peak VO2 in HFpEF [55]. Haykowsky and colleagues [56] performed magnetic resonance imaging of the thigh in twenty-three older HFpEF patients (mean age 69 years) and 15 healthy age-matched controls. This study demonstrated increased intramuscular adipose tissue area and increased adipose-to-skeletal muscle mass ratio in HFpEF patients compared to healthy controls. Both parameters were independent predictors of lower peak V̇O2. Furthermore, the natural aging process leads to skeletal muscle mass wasting, i.e., sarcopenia [57]. The compounded effect of increased body fat and reduced muscle mass is termed sarcopenic obesity [58]. As a result of these changes, older HFpEF patients typically demonstrate skeletal muscle structural abnormalities and mitochondrial dysfunction, resulting in impaired ability to utilize oxygen, and thereby contributing to exercise intolerance [57, 59].

Similar to patients with HFpEF, skeletal muscle dysfunction in HFrEF is characterized by a reduction in skeletal muscle volume/function, mitochondrial volume/function, and reduction in blood flow, contributing to exercise intolerance [53, 60, 61]. In a 1997 study, Schaufleberger and colleagues [62] performed lateral vastus muscle biopsies in 43 patients with HFrEF (mean age: 62 years) and 20 controls (mean age: 66 years). The biopsies demonstrated an increase in type II B non-oxidative fibers and a reduction in type I oxidative fibers in the HFrEF cohort. Patients with HF also showed increased baseline levels of lactate and lactate dehydrogenase activity which correlated with a decrease in aerobic exercise capacity.

### 5.2 Impaired Oxidative Capacity 

Skeletal muscles in patients with HFrEF have impaired oxidative capacity due to reduced mitochondrial volume in addition to the loss of muscle mass and impaired enzymatic activity [61]. A 2015 study by Southern and colleagues evaluated skeletal muscle oxidative capacity by measuring wrist-flexor muscle oxygen consumption using near-infrared spectroscopy in 16 HFrEF patients (average age: 65 years) and 23 controls (average age: 61 years) following wrist flexor exercises. Muscle oxidative capacity was lower in the HFrEF group (1.31 $\min^{-1}$) than in the control group (1.59 min${\mathrm{1.31}}^{-1}$). These data are consistent with prior findings in younger HFrEF patients [63].

Impaired skeletal muscle oxidative capacity is also demonstrated in elderly HFpEF patients. A 2014 study by Bhella and colleagues evaluated 11 HFpEF patients (mean age: 73 years) with CPET [15]. This study demonstrated a decreased peak V̇O2 and an increased CO/V̇O2 slope compared to healthy age-matched controls. Using ^31^phosphorous magnetic resonance spectroscopy in healthy, HFpEF, and mitochondrial disease patients, they demonstrated that the latter two groups demonstrated depleted phosphocreatine stores suggestive of impaired oxidative capacity. The pattern of impaired peak V̇O2 and increased CO/V̇O2 slopes was also similar in patients with mitochondrial disease.

### 5.3 Reduced Peripheral Perfusion Due to Impaired Vasodilation 

Older HF patients also demonstrate an impaired vasodilatory response as measured by a higher systemic vascular resistance index at peak exercise. This impaired vasodilatory response results in reduced blood flow to skeletal muscles, leading to blunted augmentation of oxygen utilization during exercise [17]. A likely contributor to the impaired vasodilatory response to exercise in elderly HF patients is impaired peripheral arterial endothelial function. Hundley and colleagues evaluated arterial dilatation following upright cycle ergometry in 10 older patients with HFrEF (mean age: 73 years), 9 with HFpEF, and 11 healthy control patients by using cardiovascular magnetic resonance imaging of the superficial femoral artery (SFA) [53]. The study demonstrated a significant decrease in flow-mediated arterial dilation (FMAD) as measured by a percent increase in SFA area in the HFrEF (4%) group as compared to either the HFpEF (12%) or control group (14%). Peak V̇O2 was positively associated with FMAD in the HFrEF cohort (*p* = 0.02) but not in the HFpEF group (*p* = 0.58).

## 6. Limitations of Existing Data

Most studies in contemporary literature on exercise intolerance in older HF populations are limited by their small sample sizes. There is also a significant under-representation of women and non-Whites in existing studies. As a result, it is difficult to generalize the conclusions of these studies to many older subgroups. There is also little homogeneity in the methodology and parameters studied across different clinical studies. For example, one study used near-infrared spectroscopy to study mitochondrial oxidative capacity [15], while another employed ^31^phosphorous magnetic resonance spectroscopy [63]. These two parameters are surrogates of oxidative capacity but are not necessarily congruent. There are also variations in exercise type, duration, and intensity across studies that further limit the generalizability of data.

Another important limitation to the existing literature is the effect of comorbidities common to older HF patients on aerobic exercise capacity. Comorbidities such as chronic renal disease, pulmonary disorders, diabetes, peripheral vascular disease, atrial arrhythmias, endocrinopathies, neurological disorders, and musculoskeletal disease affect exercise tolerance via complex pathways. Thus, the mechanisms for exercise intolerance described in this review likely vary in their importance based on a given patient’s comorbidities. Patients should therefore be evaluated in the context of their specific disease profiles.

This review does not discuss impairments in pulmonary mechanics and biochemical processes seen with both aging and HF. Both topics are also important for a global understanding of exercise intolerance in the elderly HF population but are outside the scope of the manuscript. Combined cardiopulmonary studies are needed to assess the degree to which these factors contribute to exercise intolerance in older HF patients. Finally, the role of exercise training in ameliorating the contributors to exercise intolerance in older HF patients is not addressed.

## 7. Conclusions

Heart failure is a common disorder in the elderly, leading to significant exercise intolerance. There are multiple mechanisms leading to exercise intolerance in elderly HF patients, including those due to age per se superimposed on those secondary to HF. Impairments in central parameters leading to reduced CO, including blunted heart rate, stroke volume, and blood flow distribution, are critical pathways for exercise intolerance. The elderly HF patient is also susceptible to peripheral contributors to exercise intolerance, including skeletal muscle architecture changes (i.e., increased fat mass and decreased muscle mass) as well as impaired muscle metabolism, and peripheral hypoperfusion. More clinical studies are needed in widely representative older HF populations to elucidate further these mechanisms of exercise intolerance and the role of exercise training in their treatment.

## References

[1] Virani SS, Alonso A, Aparicio HJ, Benjamin EJ, Bittencourt MS, Callaway CW (2021). Heart disease and stroke statistics - 2021 Update: A report from the American Heart Association. *Circulation*.

[2] Hunt SA, Abraham WT, Chin MH, Feldman AM, Francis GS, Ganiats TG (2005). ACC/AHA 2005 Guideline update for the diagnosis and management of chronic heart failure in the adult: a report of the American College of Cardiology/American Heart Association Task Force on Practice Guidelines (Writing Committee to Update the 2001 Guidelines for the evaluation and management of heart failure): developed in collaboration with the American College of Chest Physicians and the International Society for Heart and Lung Transplantation: endorsed by the Heart Rhythm Society. *Circulation*.

[3] Kitzman DW, Gardin JM, Gottdiener JS, Arnold A, Boineau R, Aurigemma G (2001). Importance of heart failure with preserved systolic function in patients > or = 65 years of age. CHS Research Group. Cardiovascular Health Study. *The American Journal of Cardiology*.

[4] Imamaliev QM, Azizov VA (2006). Pathophysiological characterization of isolated diastolic heart failure versus to systolic heart failure in patients with arterial hypertension. *Azerbaijan Medical Journal*.

[5] Chambers DJ, Wisely NA (2019). Cardiopulmonary exercise testing—a beginner’s guide to the nine-panel plot. *BJA Education*.

[6] Fleg JL, Lakatta EG (1988). Role of muscle loss in the age-associated reduction in VO2 max. *Journal of Applied Physiology*.

[7] Fleg JL, Morrell CH, Bos AG, Brant LJ, Talbot LA, Wright JG (2005). Accelerated longitudinal decline of aerobic capacity in healthy older adults. *Circulation*.

[8] Jackson AS, Sui X, Hébert JR, Church TS, Blair SN (2009). Role of lifestyle and aging on the longitudinal change in cardiorespiratory fitness. *Archives of Internal Medicine*.

[9] Hollenberg M, Yang J, Haight TJ, Tager IB (2006). Longitudinal changes in aerobic capacity: implications for concepts of aging. *The Journals of Gerontology Series A: Biological Sciences and Medical Sciences*.

[10] Lakatta EG (1993). Deficient neuroendocrine regulation of the cardiovascular system with advancing age in healthy humans. *Circulation*.

[11] Ho CW, Beard JL, Farrell PA, Minson CT, Kenney WL (1997). Age, fitness, and regional blood flow during exercise in the heat. *Journal of Applied Physiology*.

[12] Grimby G, Saltin B (1983). The ageing muscle. *Clinical Physiology*.

[13] Tanaka H, Dinenno FA, Monahan KD, Clevenger CM, DeSouza CA, Seals DR (2000). Aging, habitual exercise, and dynamic arterial compliance. *Circulation*.

[14] Ogawa T, Spina RJ, Martin WH, Kohrt WM, Schechtman KB, Holloszy JO (1992). Effects of aging, sex, and physical training on cardiovascular responses to exercise. *Circulation*.

[15] Bhella PS, Prasad A, Heinicke K, Hastings JL, Arbab-Zadeh A, Adams-Huet B (2011). Abnormal haemodynamic response to exercise in heart failure with preserved ejection fraction. *European Journal of Heart Failure*.

[16] Kitzman DW, Brubaker PH, Morgan TM, Stewart KP, Little WC (2010). Exercise training in older patients with heart failure and preserved ejection fraction: a randomized, controlled, single-blind trial. *Circulation: Heart Failure*.

[17] Borlaug BA, Melenovsky V, Russell SD, Kessler K, Pacak K, Becker LC (2006). Impaired chronotropic and vasodilator reserves limit exercise capacity in patients with heart failure and a preserved ejection fraction. *Circulation*.

[18] Haykowsky MJ, Brubaker PH, John JM, Stewart KP, Morgan TM, Kitzman DW (2011). Determinants of exercise intolerance in elderly heart failure patients with preserved ejection fraction. *Journal of the American College of Cardiology*.

[19] Maeder MT, Thompson BR, Brunner-La Rocca H, Kaye DM (2010). Hemodynamic basis of exercise limitation in patients with heart failure and normal ejection fraction. *Journal of the American College of Cardiology*.

[20] Esposito F, Mathieu-Costello O, Shabetai R, Wagner PD, Richardson RS (2010). Limited maximal exercise capacity in patients with chronic heart failure. *Journal of the American College of Cardiology*.

[21] Loncar G, Bozic B, von Haehling S, Düngen H, Prodanovic N, Lainscak M (2013). Association of adiponectin with peripheral muscle status in elderly patients with heart failure. *European Journal of Internal Medicine*.

[22] Roh J, Rhee J, Chaudhari V, Rosenzweig A (2016). The role of exercise in cardiac aging: from physiology to molecular mechanisms. *Circulation Research*.

[23] Fleg JL (2002). Can exercise conditioning be effective in older heart failure patients. *Heart Failure Reviews*.

[24] Rodeheffer RJ, Gerstenblith G, Becker LC, Fleg JL, Weisfeldt ML, Lakatta EG (1984). Exercise cardiac output is maintained with advancing age in healthy human subjects: cardiac dilatation and increased stroke volume compensate for a diminished heart rate. *Circulation*.

[25] Tartière-Kesri L, Tartière J, Logeart D, Beauvais F, Cohen Solal A (2012). Increased proximal arterial stiffness and cardiac response with moderate exercise in patients with heart failure and preserved ejection fraction. *Journal of the American College of Cardiology*.

[26] Wolsk E, Bakkestrøm R, Thomsen JH, Balling L, Andersen MJ, Dahl JS (2017). The influence of age on hemodynamic parameters during rest and exercise in healthy individuals. *JACC: Heart Failure*.

[27] Harber MP, Konopka AR, Undem MK, Hinkley JM, Minchev K, Kaminsky LA (2012). Aerobic exercise training induces skeletal muscle hypertrophy and age-dependent adaptations in myofiber function in young and older men. *Journal of Applied Physiology*.

[28] Puntawangkoon C, Kitzman DW, Kritchevsky SB, Hamilton CA, Nicklas B, Leng X (2009). Reduced peripheral arterial blood flow with preserved cardiac output during submaximal bicycle exercise in elderly heart failure. *Journal of Cardiovascular Magnetic Resonance*.

[29] Hollmann W, Strüder HK, Tagarakis CV, King G (2007). Physical activity and the elderly. *European Journal of Cardiovascular Prevention and Rehabilitation*.

[30] Fleg JL (2017). Exercise therapy for older heart failure patients. *Heart Failure Clinics*.

[31] Kitzman DW (2002). Pathophysiological characterization of isolated diastolic heart failure in comparison to systolic heart failure. *JAMA*.

[32] Forman DE, Clare R, Kitzman DW, Ellis SJ, Fleg JL, Chiara T (2009). Relationship of age and exercise performance in patients with heart failure: the HF-ACTION study. *American Heart Journal*.

[33] Carubelli V, Metra M, Corrà U, Magrì D, Passino C, Lombardi C (2015). Exercise Performance is a Prognostic Indicator in Elderly Patients with Chronic Heart Failure　– Application of Metabolic Exercise Cardiac Kidney Indexes Score. *Circulation Journal*.

[34] Mancini DM, Eisen H, Kussmaul W, Mull R, Edmunds LH, Wilson JR (1991). Value of peak exercise oxygen consumption for optimal timing of cardiac transplantation in ambulatory patients with heart failure. *Circulation*.

[35] Reddy YNV, Carter RE, Obokata M, Redfield MM, Borlaug BA (2018). A simple, evidence-based approach to help guide diagnosis of heart failure with preserved ejection fraction. *Circulation*.

[36] Haykowsky MJ, Brubaker PH, Morgan TM, Kritchevsky S, Eggebeen J, Kitzman DW (2013). Impaired aerobic capacity and physical functional performance in older heart failure patients with preserved ejection fraction: role of lean body mass. *The Journals of Gerontology Series a: Biological Sciences and Medical Sciences*.

[37] Haykowsky MJ, Brubaker PH, Stewart KP, Morgan TM, Eggebeen J, Kitzman DW (2012). Effect of endurance training on the determinants of peak exercise oxygen consumption in elderly patients with stable compensated heart failure and preserved ejection fraction. *Journal of the American College of Cardiology*.

[38] Nadruz W, West E, Sengeløv M, Santos M, Groarke JD, Forman DE (2017). Prognostic value of cardiopulmonary exercise testing in heart failure with reduced, mid-range, and preserved ejection fraction. *Journal of the American Heart Association*.

[39] Yan J, Gong S, Li L, Yu H, Dai H, Chen J (2013). Combination of B-type natriuretic peptide and minute ventilation/carbon dioxide production slope improves risk stratification in patients with diastolic heart failure. *International Journal of Cardiology*.

[40] Sullivan MJ, Knight JD, Higginbotham MB, Cobb FR (1989). Relation between central and peripheral hemodynamics during exercise in patients with chronic heart failure. Muscle blood flow is reduced with maintenance of arterial perfusion pressure. *Circulation*.

[41] Clark AL, Coats AJS (1995). Chronotropic incompetence in chronic heart failure. *International Journal of Cardiology*.

[42] Brubaker PH, Joo K, Stewart KP, Fray B, Moore B, Kitzman DW (2006). Chronotropic incompetence and its contribution to exercise intolerance in older heart failure patients. *Journal of Cardiopulmonary Rehabilitation*.

[43] Piña IL, Apstein CS, Balady GJ, Belardinelli R, Chaitman BR, Duscha BD (2003). Exercise and Heart Failure: A statement from the American Heart Association Committee on exercise, rehabilitation, and prevention. *Circulation*.

[44] Oxenham H, Sharpe N (2003). Cardiovascular aging and heart failure. *European Journal of Heart Failure*.

[45] Sullivan MJ, Cobb FR (1992). Central hemodynamic response to exercise in patients with chronic heart failure. *Chest*.

[46] Kitzman DW, Higginbotham MB, Cobb FR, Sheikh KH, Sullivan MJ (1991). Exercise intolerance in patients with heart failure and preserved left ventricular systolic function: Failure of the Frank-Starling mechanism. *Journal of the American College of Cardiology*.

[47] Wolsk E, Kaye DM, Komtebedde J, Shah SJ, Borlaug BA, Burkhoff D (2021). Determinants and consequences of heart rate and stroke volume response to exercise in patients with heart failure and preserved ejection fraction. *European Journal of Heart Failure*.

[48] Sandri M, Kozarez I, Adams V, Mangner N, Hollriegel R, Erbs S (2012). Age-related effects of exercise training on diastolic function in heart failure with reduced ejection fraction: the Leipzig Exercise Intervention in Chronic Heart Failure and Aging (LEICA) diastolic dysfunction study. *European Heart Journal*.

[49] Reddy YNV, Olson TP, Obokata M, Melenovsky V, Borlaug BA (2018). Hemodynamic correlates and diagnostic role of cardiopulmonary exercise testing in heart failure with preserved ejection fraction. *JACC: Heart Failure*.

[50] Little WC, Kitzman DW, Cheng CP (2000). Diastolic dysfunction as a cause of exercise intolerance. *Heart Failure Reviews*.

[51] Vigorito C, Giallauria F (2014). Effects of exercise on cardiovascular performance in the elderly. *Frontiers in Physiology*.

[52] Fujimoto N, Prasad A, Hastings JL, Bhella PS, Shibata S, Palmer D (2012). Cardiovascular effects of 1 year of progressive endurance exercise training in patients with heart failure with preserved ejection fraction. *American Heart Journal*.

[53] Hundley WG, Bayram E, Hamilton CA, Hamilton EA, Morgan TM, Darty SN (2007). Leg flow-mediated arterial dilation in elderly patients with heart failure and normal left ventricular ejection fraction. *American Journal of Physiology-Heart and Circulatory Physiology*.

[54] Haykowsky MJ, Tomczak CR, Scott JM, Paterson DI, Kitzman DW (2015). Determinants of exercise intolerance in patients with heart failure and reduced or preserved ejection fraction. *Journal of Applied Physiology*.

[55] Kitzman DW, Shah SJ (2016). The HFpEF obesity phenotype: The elephant in the room. *Journal of the American College of Cardiology*.

[56] Haykowsky MJ, Kouba EJ, Brubaker PH, Nicklas BJ, Eggebeen J, Kitzman DW (2014). Skeletal muscle composition and its relation to exercise intolerance in older patients with heart failure and preserved ejection fraction. *The American Journal of Cardiology*.

[57] Frontera WR, Hughes VA, Fielding RA, Fiatarone MA, Evans WJ, Roubenoff R (2000). Aging of skeletal muscle: a 12-yr longitudinal study. *Journal of Applied Physiology*.

[58] Upadhya B, Haykowsky MJ, Eggebeen J, Kitzman DW (2015). Sarcopenic obesity and the pathogenesis of exercise intolerance in heart failure with preserved ejection fraction. *Current Heart Failure Reports*.

[59] Conley KE, Jubrias SA, Esselman PC (2000). Oxidative capacity and ageing in human muscle. *The Journal of Physiology*.

[60] Middlekauff HR (2010). Making the case for skeletal myopathy as the major limitation of exercise capacity in heart failure. *Circulation: Heart Failure*.

[61] Mancini DM, Coyle E, Coggan A, Beltz J, Ferraro N, Montain S (1989). Contribution of intrinsic skeletal muscle changes to 31P NMR skeletal muscle metabolic abnormalities in patients with chronic heart failure. *Circulation*.

[62] Schaufelberger M, Eriksson BO, Grimby G, Held P, Swedberg K (1997). Skeletal muscle alterations in patients with chronic heart failure. *European Heart Journal*.

[63] Southern WM, Ryan TE, Kepple K, Murrow JR, Nilsson KR, McCully KK (2015). Reduced skeletal muscle oxidative capacity and impaired training adaptations in heart failure. *Physiological Reports*.

